# Experience and Impact of COVID-19 on a Newly Formed Rural University Medical Office: Survey Study

**DOI:** 10.2196/48299

**Published:** 2023-09-07

**Authors:** Mark Benton

**Affiliations:** 1 Center for Health Policy Department of Public Health University of Missouri Columbia, MO United States

**Keywords:** remote, access, COVID-19, learning, survey, office, HOPE, administration, administrative, medical education, education system, education systems

## Abstract

**Background:**

The COVID-19 pandemic had large social effects, particularly in the fields of medicine and medical education. Medical organizations in the United States operate in overlapping contexts with interrelated goals inside multiple organizations, and the context of work strongly influenced how organizations were able to respond to COVID-19 restrictions.

**Objective:**

This research examines the experience and impact of COVID-19 on the implementation of a Health Resources and Services Administration grant in a newly formed university medical office with the interrelated goals of health policy, health outreach, and medical education. The goal is to understand how COVID-19 created different experiences and challenges for leaders and their collaborators working in medical education compared to those working in public health outreach or health policy.

**Methods:**

A survey about COVID-19 opportunities and challenges was administered to work unit leaders and their project collaborators. The most common experiences and challenges are shown, direct educational and other respondents’ experiences and challenges are compared, and open-ended comment segments are analyzed.

**Results:**

Helping others adjust to digital work, remoteness, and coordination were common experiences during COVID-19. Common challenges include coordination and an inability to make comparisons to previous program years. On average, respondents had 11.3 (SD 7.8) experiences and 8.3 (SD 6.9) challenges considered in the survey. While all units were influenced by COVID-19 restrictions, medical education units had more experiences and challenges. Those involved directly in medical education experienced 69% (18.6/27) of their possible experiences and 54% (14.7/27) of their possible challenges on average compared to 35% (7/20) and 21% (4.2/20) among other respondents (*P*<.001). COVID-19 restrictions increased the complexity of project work and presented challenges, especially in terms of coordinating responses and access to locations.

**Conclusions:**

The findings suggest that COVID-19 made the overall administration of programs more complex and drew attention from other medical and public health programs. While remoteness is appropriate for some medical education tasks, it is less appropriate for clinical learning. Remoteness presents an especially large challenge to clinical education. Employees now have expectations for remoteness to be built into programs and workplaces. Program administrators will have to integrate remoteness’ benefits and drawbacks into their organization for the foreseeable future.

## Introduction

### Background

On March 11, 2020, the World Health Organization declared COVID-19 to be a pandemic, defined as “the worldwide spread of a new disease” that has the potential to infect many people [1]. Responses such as school closures, shutdowns, and stay-at-home orders were implemented as vital public health interventions to the threat that COVID-19 placed on communities and individuals, especially for those most susceptible to the disease [2,3]. It would also be wrong to say that these interventions did not influence medicine and medical education.

Starting March 11, 2020, the University of Missouri ceased in-person learning “out of an abundance of caution,” with initial plans to transition to remote education until March 30, 2020. The university would not actually resume in-person education in a way that reflected prior operations until January 2022. As mandatory remote work faded, many work units found themselves functional while maintaining remote work, while others were eager to return to in-person work.

The author participated in a Health Resources and Services Administration (HRSA) grant–funded project with the Office of Health Outreach, Policy, and Education (HOPE) at the University of Missouri serving evaluation support functions. The Office of HOPE is an office at the University of Missouri that oversees and coordinates work units involved in telehealth, health policy, medical education in rural Missouri, health outreach, and health professionals’ continuing education. The organization of HOPE can be seen in Multimedia Appendix 1. HOPE especially participates in rural medical education in Missouri, with a clerkship for rural medical students, a pipeline program to expose students to rural medicine, and scholarship programs [4]. HOPE subunits existed prior to the pandemic, but HOPE itself was formed simultaneously with the rise in relevance of COVID-19 in the United States.

Medical education experienced numerous challenges from COVID-19 and prevention measures. The normal routines of medical education were disrupted at a global level and medical education programs had to adapt [5]. Many medical education programs moved to remote education and telemedicine [6-8], including those in HOPE. The transition to remoteness was a public health precaution with benefits like improved safety, convenience, and access. At the same time, remote work has drawbacks such as stunted career trajectories [9], reduced camaraderie [10], and changing team dynamics [11]. While remoteness will remain relevant for the foreseeable future, that fact will have different implications for different kinds of medical organizations, and managers will have to consider the suitability of remoteness for the tasks at hand in mind.

### Study Objectives

The goal of this study is to understand how employees in different departments of a university’s rural medical office experienced and were challenged by COVID-19 and if those experiences and challenges were different depending on whether they provide direct educational services.

## Methods

### Study Population and Setting

The research consists of a survey of HOPE unit leaders. A list of 14 people in HOPE unit leadership positions was created, and they were reached out to with a recruitment email. After contextualizing the survey as applying to the HRSA-funded projects, two requests were made to (1) take the survey and (2) distribute the survey to those they identified as crucial partners for working on their HRSA-funded project. A sample email was included for unit leaders to use in contacting others to reduce the burden of composing the second email. This kind of exponential nondiscriminative snowball sampling has been used in other research to study illness and work [12], mental health [13], and interprofessional care [14].  This approach is helpful when the people being surveyed are in a network that is not completely known to researchers [15,16]. The survey opened on July 12, 2022, and closed on August 17, 2022. The survey received a total of 25 responses overall.

### Survey Design

The survey was designed to measure the experiences of COVID-19 and how those experiences challenged work. To accomplish this, web searches were performed with variations of the phrase “COVID-19 Professional Impact Form.” Twenty-nine performance review lists, templates, and suggested areas of impact from universities were identified to understand the many ways in which COVID-19 could have influenced projects’ work. Numerous organizations noted the professional and career implications COVID-19 measures posed [17-23]. These documents were from universities across the United States and applied to faculty, administrators, and other employees.

From an inductive examination of documents, 10 areas that could influence work were identified: (1) access to locations, (2) changing work duties, (3) caregiving or home disruption, (4) technology challenges, (5) event and travel cancellation, (6) mentoring or service, (7) illness, (8) funding, (9) supply chains and logistics, and (10) other idiosyncratic areas. Many documents also provided space for people to describe their pandemic experiences with an open-ended response. Questions for the survey were phrased to measure COVID-19 experiences and the challenges of these experiences for work. Survey questions on these topics are in Multimedia Appendix 2.

### Survey Organization

**Figure 1:**
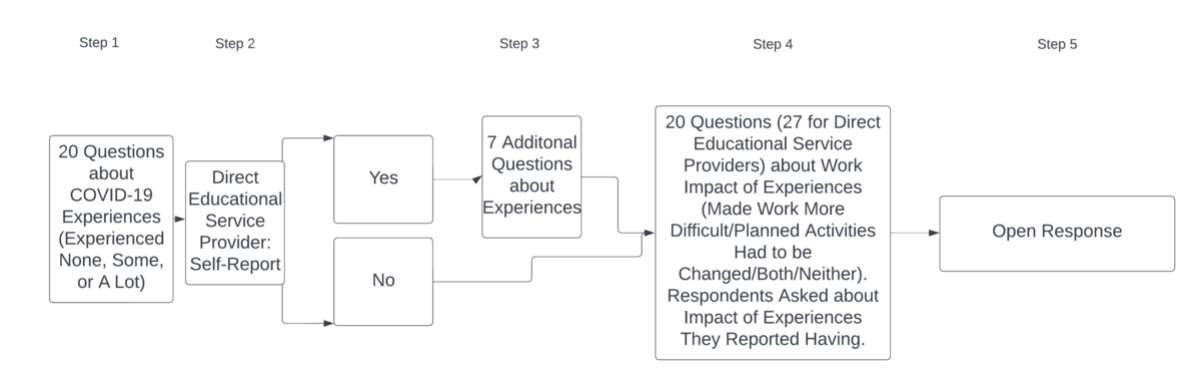
Survey organization.

The survey had 5 main steps. In step 1, respondents were asked 20 questions about their experiences of COVID-19 with 2 questions for each of the 10 areas. Areas could have been experienced “none,” “some,” or “a lot.” Language use for response options was informal to cater to work unit leaders and their diverse partners [24-27]. During the survey design process, some questions were found to be most appropriate for medical educators but would be irrelevant for noneducators. To address this in a way that minimized the survey burden for respondents, in step 2, respondents were asked if they were delivering direct educational services, defined as working in a program that teaches students or learners or very closely supporting those who do so. Some examples of those in direct educational services could include those providing continuing medical education to health professionals or health education to medical students, while those not providing direct educational services could work in areas like procurement, health outreach, or health policy. Those who responded “yes” answered 7 additional questions about their experiences in step 3, and other respondents advanced to step 4 immediately. In step 4, participants were asked about the challenges their experiences created, for the experiences they had reported having “some” or “a lot.” Respondents were not asked if things they did not experience created challenges to minimize the survey burden. Finally, in step 5, respondents described the most important ways that COVID-19 influenced their work in their own words, both positively and negatively. Figure 1 visualizes the organization of the survey.

### Survey Reporting

This survey is reported following the Checklist for Reporting Results of Internet E-Surveys (CHERRIES) [28]. The checklist is shown in Multimedia Appendix 3.

### Quantitative Methods: Descriptive and Statistical Analysis

Descriptive statistical results of survey responses to questions about experiences and challenges of COVID-19 are presented. Respondents answered questions about their experiences: if the experience was something they did not experience, experienced some, or experienced a lot. An experience was considered to have created a challenge if respondents indicated that the experience impacted HRSA-sponsored work in a way that “made work more difficult,” necessitated that “planned activities had to be changed,” or both. This descriptive analysis is conducted for respondents in aggregate and separately for respondents providing direct educational services to understand common experiences and challenges. Following that, direct educational service respondents are compared to others in terms of experiences and challenges with statistical methods, using 2-tailed *t* tests to compare the differences in the percentage of experiences and challenges [29,30].

### Qualitative Methods

#### Overview

To analyze open-ended responses, each response was organized into spreadsheet columns labeled with code names. Code names were created inductively as each response was sorted into columns. This helped to understand the codes in the data from the beginning of the analysis process, which made clear that each response could discuss more than 1 aspect of how COVID-19 influenced work. Each response was then transferred from the spreadsheet to a word-processing document for analysis on a subresponse level.

Text segments were coded. Text segments are defined as portions of responses that fully communicate an idea, which may be less than a single sentence, a single sentence, or multiple sentences. Scholars have referred to these whole-idea units with names like *lexia* [31-33] or *ideograph* [34-36]. The goals of the survey were to understand the COVID-19 experiences and the challenges as they related to HRSA project work, so the aspects of responses that did not relate to those topics were not coded. Based on this analysis, 5 themes were identified.

#### Working Environment

Segments were coded within this theme when they referred to how the work arrangements created by COVID-19 responses led to less face-to-face contact, socializing, interpersonal knowledge, and interpersonal learning among project members.

#### Medical Availability

Segments were coded in this theme when they communicated that people who were required to complete or deliver the project were more difficult to recruit or did not have time for participation because of changes in conditions caused by COVID-19 responses.

#### Complexity

Segments were coded in this theme when they communicated that COVID-19 changed the nature of the work being done and how it was being done. This encompasses factors including implementing and accomplishing new digital work processes, changes in work practices and procedures, and evaluation challenges.

#### Low Impact

Segments were coded in this theme when they communicated that changes in work conditions caused by responses to COVID-19 did not have much influence on how work occurred.

#### Positive Impact

Segments were coded in this theme when they communicated that changes in conditions created by COVID-19 and the response to it resulted in a positive change in work life.

Furthermore, several steps were taken to improve research trustworthiness [37,38]. To improve credibility, research participants were member checked by providing team leaders with a presubmission copy of an initial research manuscript and a synchronous digital presentation of findings. No objections to findings or presentation of other facts were made. To help readers make decisions about transferability, the introduction includes a description of the context of the program where the study occurred. To help improve dependability, the findings were checked with a research supervisor and the overall HRSA project principal investigator, who noted that the findings comported with their experiences. Finally, to improve confirmability, the researcher took no stance for research on the appropriateness of COVID-19 work restrictions or the continuing of remote work in general. The researcher has an individual preference for remote work but recognizes a diversity in preferences and the inappropriateness of some tasks for remote work. Remoteness, its effects, and its implications must be examined empirically.

### Ethics Approval

This project (2092115) was approved and granted exempt status by the institutional review board at the University of Missouri on July 7, 2022.

## Results

### Descriptive Quantitative Results

On average, respondents reported 11.3 (SD 7.8) of the experiences that were considered in the survey. The most common experience (19/25, 76% of respondents indicated they experienced it “some” or “a lot”) was helping others adjust to digital work. The second most common experience (18/25, 72%) was that changes in programs made comparisons to previous years impossible, with the third most common experience (16/25, 64%) being a tie between (1) having reduced access to facilities and (2) having to hold additional meetings to coordinate an organizational response to COVID-19.

Respondents indicated that on average 8.3 (SD 6.9) of the experiences considered created challenges. An experience was considered to have created a challenge if respondents indicated that the experience challenged HRSA-sponsored work in a way that “made work more difficult,” necessitated that “planned activities had to be changed,” or both. Respondents experienced on average 4.0 (SD 4.4) challenges that required changes, 3.7 (SD 4.3) that made work more difficult, and 0.6 (1.9) that both made work more difficult and required changes. The most common (15/25, 60%) COVID-19 challenge was the necessity of additional meetings to coordinate organizational responses to COVID-19. The second most common (14/25, 56%) challenge was that changes in programs made comparisons to previous years impossible. The third most common challenge (13/25, 52%) was tied between (1) reduced access to facilities and (2) the development of new technologies and platforms for remote service delivery.

Of those who responded to the question (22/25, 88%), 11 respondents (11/22, 50%) reported that they were involved in a project that was delivering direct educational services or closely supporting those delivering direct educational services. There was homogeneity within direct educational service respondents’ experiences. The most common COVID-19 experiences (11/11, 100%) for educational respondents were a tie between (1) having reduced access to facilities, (2) additional meetings to coordinate organizational responses to COVID-19, (3) helping others adjust to digital work, and (4) cancellation of travel. The set of second most common experiences for respondents in educational projects (10/11, 91%) included (1) workload increases to develop plans for closing and reopening of facilities or locations; (2) changes in official responsibilities; (3) development of new technologies and platforms for remote service delivery; (4) increased voluntary workplace services, not covered by official responsibilities, to maintain organizational operations; (5) changes in programs made comparisons to previous years impossible; (6) restrictions impacted the ability to collaborate with partners; (7) course delivery required changes for blended learning; and (8) course delivery required changes for digital student engagement. The third most common (9/11, 82%) experience among educators was restricted access to supplies.

Each experience could have created challenges for those in educational projects. The most common challenge (11/11, 100%) was a tie between (1) reduced access to facilities and (2) additional meetings to coordinate organizational response to COVID-19. The second most common challenges (10/11, 91%) were (1) restrictions impacting the ability to work with partners, (2) the development of new technologies and platforms for remote service delivery, and (3) course delivery required changing for digital student engagement. The third most common (9/11, 82%) impacts included (1) changes in official workplace responsibilities, (2) cancellation of travel, and (3) that course delivery required changing for blended learning. Educational respondents reported that on average 14.7 (SD 3.2) of their experiences created challenges. Of experiences that created challenges in direct service educational projects, on average 7.4 (SD 4.2) required that planned activities be changed, 6.1 (SD 4.4) made work harder, and 1.2 (SD 2.7) both required changing planned activities and making work harder.

### Statistical Quantitative Results

Those who identified as being in educational projects reported a higher percentage of experiences and a higher percentage of challenges. Comparing respondents who reported being in direct educational services to those who did not require analyzing the percentage of the total possible experiences and challenges because direct educational service projects had 7 more possible experiences and challenges overall.

Those in projects delivering educational services reported on average experiencing 69% (18.6/27) of their possible experiences compared to 35% (7/20) among other respondents. An independent 2-tailed *t* test performed in Stata (Stata Corp) indicates a difference of about 35% between the 2 (*P*<.001). Those in educational service projects reported 54% (14.7/27) of the total possible challenges, while others reported 21% (4.2/20). An independent 2-tailed *t* test performed in Stata indicates a difference of about 37% between the two (*P*<.001). Table 1 summarizes these results.

**Table 1:** Statistical quantitative findings.

	Aggregated number of experiences, mean	Educational respondents (N=27), n (%)	Noneducational respondents (n=20), n (%)	*P* value
Experiences	11.3	18.6 (69)	7 (35)	<.001
Challenges	8.3	14.7 (54)	4.2 (21)	<.001

### Open-Ended Response Results

In total, 37 text segments were coded in 13 responses from those that left a qualitative comment, leading to a mean of about 2.8 coded segments per comment. Seventeen (46%) were coded in complexity, 7 (19%) segments were coded in working environment, 7 (19%) were coded in medical availability, 3 (8%) were coded in low-impact, and 3 (8%) were coded in positive impact. 

Open-ended response data suggest that the most prominent influence of COVID-19 on HRSA projects was making them more complex. Some examples of segments coded with the complexity theme include the following: (1) “We had medical students all over the state and had to keep track of COVID outbreaks and PPE at all of our sites to ensure student safety and it was different at each site.” (2) “We no longer had face-to-face interactions with students and stakeholders, couldn’t host planned meetings, weren’t allowed to work on-site, and had to change everything about our work environment.” (3) “Adaptation took quite a while, and when offices re-opened, adaptation was required again.”

The second most common theme was medical availability. Projects experienced challenges in recruitment and participation, especially projects that required medical faculty development or participation. Some segments for this theme include the following: (1) “I believe that COVID had a massive impact on the ability for rural health care providers to take time out of their week/month for educational opportunities.” (2) “While a number of people seemed willing to contribute to the creation of our project, most were faced with significant time constraints due to patient volume and staff shortages. It likewise became challenging to produce materials on topics OTHER than COVID, as that was on everyone’s mind.” (3) “The increased workload of our target audience made this participation fall to low priority when their time was pulled in so many other directions.”

The third most common theme was the working environment. Projects were at times challenged by a change to digital work as it related to working relationships. Some segments include the following: (1) “All staff engagement activities were delivered virtually and that made it more difficult to engage 60+ individuals and to create a sense of community across the HOPE units.” (2) “My mind goes primarily to the challenges of building new professional relationships under the conditions of remote and/or hybrid work.” (3) “I was attempting to manage/supervise many without being able to meet them face to face or travel which made it difficult to know and learn.” (4) Some also responded that COVID-19 had a low impact on their work, for example, 1 segment said, “It is difficult for me to say that COVID meaningfully impacted my HRSA work directly.” Others pointed out that COVID-19 could even create positive changes, saying that “It did accelerate the idea that more training is needed in rural communities on how to best use telehealth” and that “we now have incontrovertible evidence that working remotely is possible and successful.”

## Discussion

### Principal Findings

This research identified that during COVID-19 restrictions, respondents to a survey in a newly formed medical office most commonly experienced helping others adjust to digital work, difficulties comparing programs to the past, reduced access to facilities, and increased meetings to coordinate responses. Respondents were most challenged by additional coordinating meetings, program comparisons, reduced access to facilities, and the implementation of new technology. Respondents in direct educational service programs expressed similar challenges and had more experiences and challenges overall than others. Open-ended response data suggest that challenges arose from a need for programs to be more complex, and that medical attention to COVID-19 drew attention from other medical programs in a way that made work more challenging.

### Comparisons to Previous Work

One large challenge for respondents was a lack of access to facilities for in-person programming. Those who delivered remote medical education during COVID-19 are likely better positioned to deliver it in the future than they would be otherwise [39,40]. At the same time, research [41-43] highlights the difficulties remote medical education poses and that many medical education programs were challenged to deliver hands-on clinical education remotely. While classroom-based learning can be accomplished in a reduced form remotely, clinical medical education is extremely challenging to accomplish remotely.

Eight percent of segments referred to positive changes created by COVID-19, and all of those referred to how remoteness—either for respondents or for their clients—is not an insurmountable challenge and could be preferable to in-person work. Remote work is not advised for all employees, as some perform better under in-person conditions [44] and others prefer remote work but would have improved careers with in-person work [9]. Survey respondents indicated in open responses that new member socialization is difficult in remote conditions. Positive implications of remote work include increased productivity, flexibility, and autonomy and negative implications include isolation, blurred work and home boundaries, and decreased visibility of employees’ productive activities [10].

Regardless of preferences, managing medical programs for the foreseeable future will likely entail remote work. The environment in which organizations exist has expectations for flexible work locations. Digital communications will supplant in-person communications. Expectations for the future are that face-to-face communications may be more spontaneous than before. For communications about large changes in organizations, communication channels that allow for 2-way communication are likely most appropriate because they provide opportunities for questions and feedback. On the other hand, very complex issues may be poorly handled via digital methods because minor clarifications or definitional issues are difficult to resolve through this kind of web-based call and response, and a more immediate form of communication like telephone can be more appropriate [45].

### Limitations and Strengths

For the first limitation, participants’ demographic data were not collected to protect their privacy. This creates limitations because responses between groups except for participation/nonparticipation in direct educational service work cannot be compared. Additional relationships between experiences or challenges and other characteristics may have arisen with additional data. At the same time, some demographic characteristics may have been singular and would have threatened participant privacy in the project. Second, while feedback was obtained to buttress trustworthiness, the research, analysis, and reporting of these data were conducted by a single researcher. Additional researchers may have been able to produce additional results, create a superior survey design, or improve the overall quality of the research. Third, work unit leaders were asked to distribute surveys to crucial partners, but it is uncertain how many partners were solicited or could have been solicited, and thus response rates cannot be calculated. The administration of the survey to partners was left in part in the hands of leaders because they knew their crucial partners best, but that creates uncertainty about how many partner respondents were and could have been solicited. Finally, the sample was a convenience sample, and this may bias results in uncertain directions were they to be applied to other medical offices. Readers should carefully consider if insights accurately transfer to their organization prior to integrating them, as not all organizations face the same kinds of conditions as the sampled organization.

One strength of this study is that it considers different kinds of university medical office programs together rather than separately. Other studies often focus on only medical education or the administration of other kinds of programs. Comparing the experiences and challenges of the 2 groups acknowledges the interconnected nature of medical, medical education, and public health programs while also highlighting their differences.

### Conclusions

COVID-19 created challenges for public organizations, but not all challenges were experienced equally and not all contexts were presented with the same level of challenge when faced with the same issues. Different organizations exist in different contexts and work in different areas, and this variation may have created differences in how the COVID-19 pandemic was experienced and the challenges it created. This analysis indicates that medical education organizations had more experiences because of COVID-19 than others and that those experiences created more challenges than other kinds of university medical projects. COVID-19 also made work more complex by requiring additional time to plan projects around the changes COVID-19 required like remote delivery, blended learning, and economic uncertainty. While remote work and delivery can be positive, and some medical educational tasks are amenable to remote learning, remoteness also created challenges for organizations. Hybrid (remote/in-person) working arrangements will remain for the foreseeable future. For managers, challenges for the foreseeable future will entail managing these arrangements, identifying what is possible and advisable to conduct digitally and what is not, and ensuring socialization in work environments.

## Appendix

Multimedia Appendix 1 Organization of the Office of Health Outreach, Policy, & Education (HOPE).

Multimedia Appendix 2 Possible experiences and impacts in survey.

Multimedia Appendix 3 CHERRIES (Checklist for Reporting Results of Internet E-Surveys) checklist.

## Abbreviations

CHERRIES: Checklist for Reporting Results of Internet E-Surveys
HOPE: The Office of Health Outreach, Policy, and Education
HRSA: Health Resources and Services Administration

## Data Availability

The data sets generated and analyzed during this study are available from the corresponding author upon reasonable request.
