# Supplementary figures and images for: Experience and Impact of COVID-19 on a Newly Formed Rural University Medical Office: Survey Study
Source: JMIR Form Res. 2023 Sep 7;7:e48299. doi: 10.2196/48299 (PMC10514764; doi:10.2196/48299)

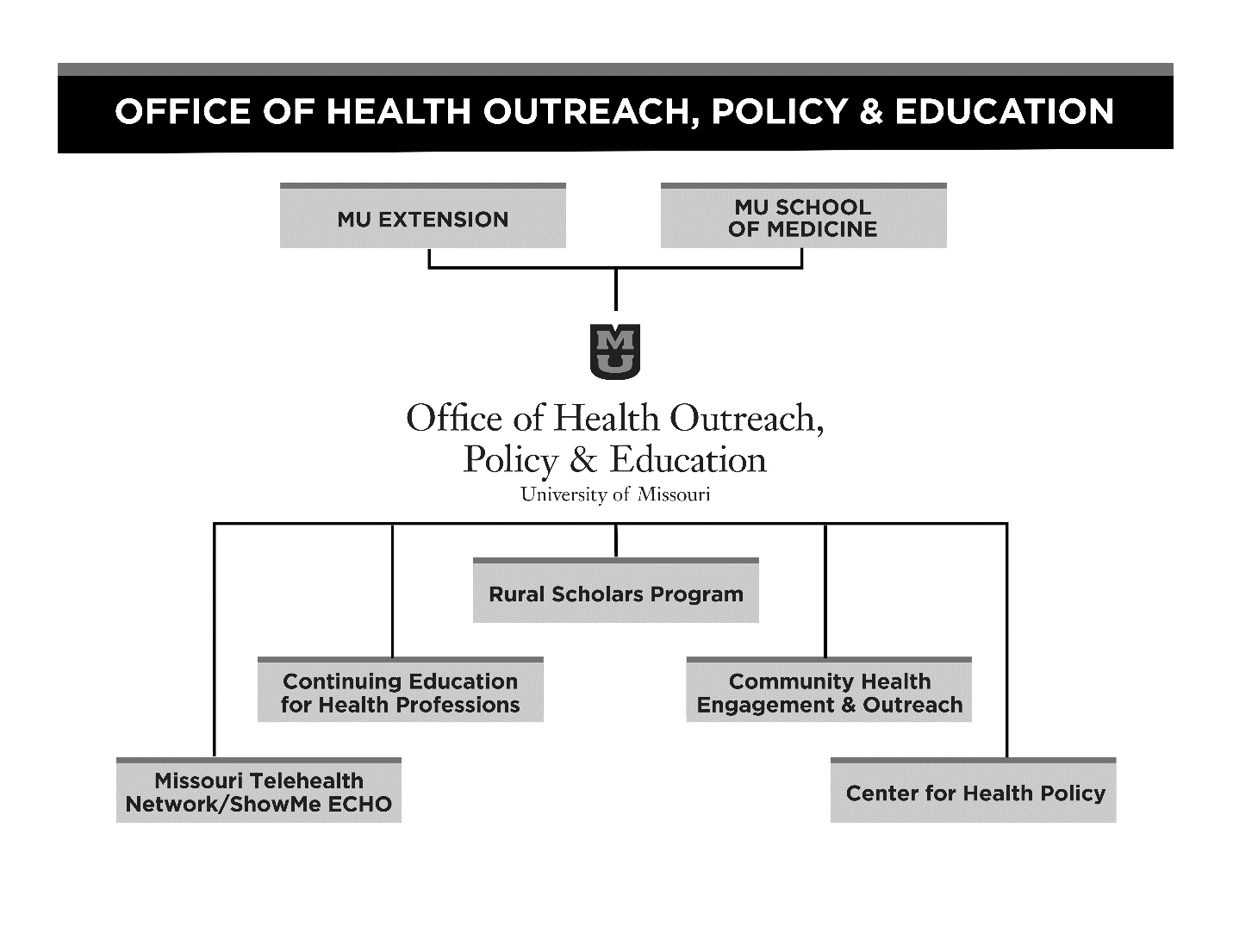


**Organization of the Office of Health Outreach, Policy, & Education (HOPE).**

Supplement: Multimedia Appendix 1 [file formative_v7i1e48299_app1.docx]
