# Supplementary material for: Experience and Impact of COVID-19 on a Newly Formed Rural University Medical Office: Survey Study
Source: JMIR Form Res. 2023 Sep 7;7:e48299. doi: 10.2196/48299 (PMC10514764; doi:10.2196/48299)
Supplement: Multimedia Appendix 2 [file formative_v7i1e48299_app2.docx]

| **Possible experiences for all respondents** |
| --- |
| - Reduced access to facilities or locations required to complete activities |
| - Workload increases to develop plans for closing and re-opening of facilities or locations |
| - Changes in official workplace responsibilities |
| - Decreases in my overall weekly work hours |
| - Additional meetings to coordinate organizational response to COVID-19 |
| - Increased caregiving (childcare, eldercare, etc.) responsibilities |
| - Direct responsibility for overseeing a child's education |
| - Development of new technologies and platforms for remote service delivery |
| - Needed time for completing online certification/learning new tools |
| - Cancelation of travel |
| - Increased voluntary workplace services, not covered by official responsibilities, to maintain organizational operations |
| - Helping others adjust to online work |
| - Pre-existing conditions hampered ability to work during COVID-19 pandemic |
| - Illness with COVID-19 |
| - Using funding for PPE or other precautionary measures related to COVID-19 |
| - Unplanned restrictions in use of funds |
| - Delays in the review and/or production of publications or other scholarly products |
| - Restricted access to supplies/disruption in supply chains disrupted purchase plans |
| - Changes in programs made comparisons to previous programs impossible |
| - Restrictions impacted ability to collaborate with stakeholders |
| **Possible experiences for those identifying as providing direct medical educational services** |
| - Cancellation of in-the-field courses/demonstrations/programs |
| - Spending increased time addressing safety needs related to clinical training (students, residents, interns) |
| - Spending increased time addressing safety needs related to client/patient interactions |
| - Time spent on efforts to obtain training on remote teaching techniques |
| - Course delivery required changes for blended learning |
| - Course delivery required changing for virtual student engagement |
| - Interruption of clinical teaching and supervision of internships; the need to revise how those programs are designed and delivered |
|  |
|  |
|  |
|  |
|  |
| **Open Ended Response** |
| Finally, we'd like to give you an opportunity to describe any ways that COVID-19 influenced your work on MSEP projects in your own words. Think about the time since March 2020, how much has changed since then, and how you have had to respond in your organization.  Over the course of that time, how did COVID-19 and the response to COVID-19 influence what happened in your HRSA-funded MSEP project? For example, many courses are now available online in a way that is both accessible and engaging to many people.  On the other hand, changes in how courses are delivered may have also created challenges for completing work. COVID-19 presented numerous challenges and changes. Do you have examples of how you or your organization responded to COVID-19 and how that influenced what happened in the HRSA-funded MSEP projects you worked on?  Please describe the most important ways that COVID-19 influenced your work on MSEP projects. Write as much and in as much detail as you would like. |

Note: Participation in medical education projects was determined as including those who responded yes to the question: “Were you involved in delivering direct educational services or closely supporting those delivering direct educational services in the HRSA-funded MSEP? This includes working in programs that involve teaching students or learners.”

**Possible Experiences and Impacts in Survey.**
