# Supplementary material for: Experience and Impact of COVID-19 on a Newly Formed Rural University Medical Office: Survey Study
Source: JMIR Form Res. 2023 Sep 7;7:e48299. doi: 10.2196/48299 (PMC10514764; doi:10.2196/48299)
Supplement: Multimedia Appendix 3 [file formative_v7i1e48299_app3.docx]

|  | **Checklist for Reporting Results of Internet E-Surveys (CHERRIES)** | |
| --- | --- | --- |
| ***Item Category*** | ***Checklist Item*** | ***Explanation*** |
| **Design** |  |  |
|  | Describe survey design | Sample included university medical office work unit leaders and their most crucial project partners. Exponential non-discriminatory snowball sampling was used by asking work leaders to recruit partners. Survey first asks about experiences, then if respondent was involved in the direct education of students. Those involved are asked about additional experiences. All respondents were then asked about if the experiences they had created challenges. All respondents were then asked for qualitative information. |
| **IRB (Institutional Review Board) approval and informed consent process** |  |  |
|  | IRB approval | This project (# 2092115) was approved and grated exempt status by the institutional review board at the University of Missouri on July 7 2022. |
|  | Informed consent | Informed consent was provided at the beginning of the voluntary survey, with no penalty for dropping out at any time or not answering any specific question. Respondents were told the survey could take up to 20 minutes. Respondents were told the purpose of the study was to help understand how COVID-19 restrictions impacted work activities, who the investigator was, how to contact IRB or the investigator with any questions or issues. Informed consent included that no individual identifiers would be collected. This informed consent was approved as part of the University IRB process. |
|  | Data protection | No personal identifiers were collected. Data is stored in an online repository approved by institutional IRB |
| **Development and pre-testing** |  |  |
|  | Development and testing | Feedback was sought from supervisors and the overall project investigator. The survey was piloted through two processes, with 4 students then again with 100 respondents using Amazon’s Mechanical Turk sample. |
| **Recruitment process and description of the sample having access to the questionnaire** |  |  |
|  | Open survey versus closed survey | This was conducted as an open survey. |
|  | Contact mode | Contact with project leaders was made electronically and included a survey link. Contact with project leader partners was left to the discretion of project leaders, but were advised to contact over email and were provided with a recommended partner recruitment email template. |
|  | Advertising the survey | The survey was not advertised, but was sent directly to key respondents. |
| **Survey administration** |  |  |
|  | Web/E-mail | Survey was posted on a website |
|  | Context | Survey website was Qualtrics |
|  | Mandatory/voluntary | Survey was voluntary |
|  | Incentives | No incentives were offered |
|  | Time/Date | The survey opened on July 12th, 2022 and closed on August 17th, 2022 |
|  | Randomization of items or questionnaires | No item or question randomization occurred |
|  | Adaptive questioning | In the challenges section of the survey, respondents were only asked about challenges from experiences they reported having had in an earlier section of the survey. |
|  | Number of Items | No more than 10 items per page, in a matrix table |
|  | Number of screens (pages) | 9 screens |
|  | Completeness check | No completeness checks were performed prior to submitting the survey. |
|  | Review step | Respondents could review or change answers by using a back button. |
| **Response rates** |  |  |
|  | Unique site visitor | Unable to report |
|  | View rate (Ratio of unique survey visitors/unique site visitors) | Unable to report |
|  | Participation rate (Ratio of unique visitors who agreed to participate/unique first survey page visitors) | Unable to report |
|  | Completion rate (Ratio of users who finished the survey/users who agreed to participate) | Unable to report |
| **Preventing multiple entries from the same individual** |  |  |
|  | Cookies used | No cookies were used |
|  | IP check | No IP addresses were used, in line with IRB review of avoiding personal identifiers |
|  | Log file analysis | No other techniques were used. |
|  | Registration | No applicable |
| **Analysis** |  |  |
|  | Handling of incomplete questionnaires | All questionnaires passing informed consent were analyzed |
|  | Questionnaires submitted with an atypical timestamp | No time frame cut off was used |
|  | Statistical correction | No statistical correction was employed. |

**CHERRIES Checklist**
